# Local Treatment Efficacy for Single-Area Squamous Cell Carcinoma of the Unknown Primary Site

**DOI:** 10.3390/curroncol30100674

**Published:** 2023-10-20

**Authors:** Tomoko Kurita, Mayu Yunokawa, Yuji Tanaka, Kota Okamoto, Motoko Kanno, Atsushi Fusegi, Makiko Omi, Sachiho Netsu, Hidetaka Nomura, Akiko Tonooka, Hiroyuki Kanao

**Affiliations:** 1 Department of Obstetrics and Gynecology, School of Medicine, University of Occupational and Environmental Health, Kitakyushu 807-8555, Japan; hiroyuki.kanao@jfcr.or.jp; 2 Department of Gynecologic Oncology, Cancer Institute Hospital of the Japanese Foundation for Cancer Research, Tokyo 135-8550, Japan; mayu.yunokawa@jfcr.or.jp (M.Y.); yujit@belle.shiga-med.ac.jp (Y.T.); kota-okamoto@med.mie-u.ac.jp (K.O.); motoko.kanno@jfcr.or.jp (M.K.); atsushi.fusegi@jfcr.or.jp (A.F.); makiko.omi@jfcr.or.jp (M.O.); sachiho.netsu@jfcr.or.jp (S.N.); hidetaka.nomura@jfcr.or.jp (H.N.); 3 Division of Pathology, Cancer Institute, Japanese Foundation for Cancer Research, Tokyo 135-8550, Japan; akiko.tonooka@jfcr.or.jp

**Keywords:** squamous cell carcinoma, cancer of unknown primary site, SCCUP, local treatment, prognosis

## Abstract

The prognosis for cancer of unknown primary site (CUP) is poor, and squamous cell carcinoma of the unknown primary site (SCCUP) is a rare histological type. CUP is often treated with aggressive multimodal treatments, while the treatment of single-area localized CUP remains controversial. We retrospectively reviewed the medical records of patients with CUP. SCCUP in women was classified according to several definitions. Based on the histologic type and site, they were classified into favorable and unfavorable subsets. We further divided SCCUP into two types (single and multiple areas) and reviewed treatment and efficacy. Among the 227 female CUP patients, 36 (15%) had SCCUP. The median age was 59.9 years (range, 31–90 years). Most patients (61.1%) had a good performance status. Of the SCCUP patients, 22 had cancer in a single area, and 14 in multiple areas. Single-area SCCUP was further divided into favorable (16 cases) and unfavorable subsets (6 cases). In the favorable subset, local treatment was predominant, and almost all cases had a good prognosis. Even in the unfavorable subset, local therapy was combined with systemic chemotherapy in only two cases, and four cases showed no recurrences. Local treatment may be effective for single-area SCCUP, even in the unfavorable subset.

## 1. Introduction

Cancer of unknown primary site (CUP) is defined as metastatic cancer without an identifiable primary tumor on systemic examination [1], and a heterogeneous neoplasm of different origin from the metastatic site. The prognosis for CUP is extremely poor, with a median survival of only 6–9 months [2,3,4]. Squamous cell carcinoma of the unknown primary site (SCCUP) is even rarer (5–10% of CUP cases) [5,6] and is less sensitive to anticancer drugs than adenocarcinoma of the unknown primary site.

The treatment strategies for CUP depend on whether it is classified as a favorable or unfavorable subset. The favorable subset in SCCUP is limited to tumors in the cervical or inguinal lymph node region, and local treatment is recommended, including surgery and radiotherapy, whereas systemic chemotherapy is recommended for the unfavorable subset [6,7,8]. Radiotherapy is often effective in cases of SCC, and it may also be sufficiently effective in single-area SCCUP, even in the unfavorable subset, and this has not been investigated.

Although gynecologists often treat SCC in cervical cancer, various clinical departments may be responsible for the treatment of SCCUP. Treatment decisions will often be challenging when SCC is localized in the pelvis and the primary site is unknown. The rarity and disease specificity of CUP make it difficult to conduct large-scale clinical trials, and no standard treatment has been established. In this present study, we investigated the efficacy of local treatment for single-area SCCUP in females.

## 2. Materials and Methods

A total of 227 female CUP patients were treated at our hospital between 2010 and 2019. The study was approved by The Cancer Institute Hospital of JFCR Review Board (Institutional Review Board) and was conducted in accordance with the relevant guidelines and regulations of the Institutional Review Board.

**Figure 1:**
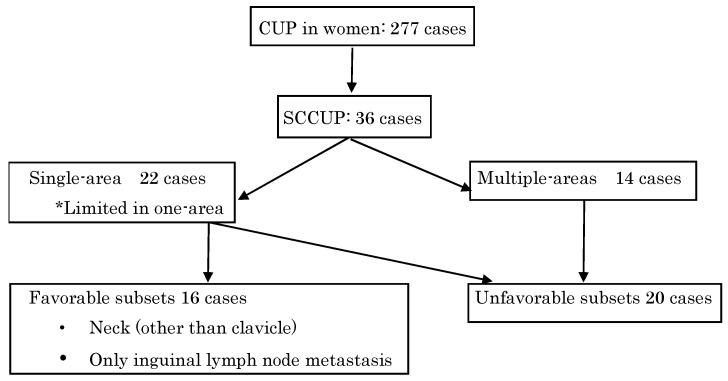
CONSORT flow diagram.

All patients, except those who visited for a consultation/second opinion, underwent a basic evaluation consisting of medical history, physical examinations, laboratory tests, and imaging studies. Magnetic resonance imaging of the pelvis and computed tomography of the upper abdomen and chest were performed to evaluate the tumors, regional extent of the disease, lymph node status, and distant metastasis. Laryngoscopy and gastrointestinal endoscopy were also performed. Pathological materials were obtained via excision or needle biopsy and were carefully assessed. Pathological materials obtained at a previously visited hospital were also reviewed again by the pathological division of our institution. Only cases of SCCUP were selected, and the patient background, treatment, and prognosis were examined. Figure 1 illustrates the flow of patients included in this study.

Patients were divided into two groups: single-area and multi-area. Single-area type cases was defined as SCCUP lesions that remained in one area. Furthermore, patients with single-area SCCUP were divided into favorable and unfavorable subsets. Based on previous reports, the cases described below were defined as a favorable subset (SCC involving cervical lymph nodes, SCC involving inguinal lymph nodes, and adenocarcinoma involving only axillary lymph nodes), and the other cases were defined as an unfavorable subset [9]. The favorable subset with single-area SCCUP represents metastasis to cervical (excluding clavicular) or inguinal lymph nodes. Other single-area SCCUP cases were classified as an unfavorable subset.

The physicians determined the treatments for individual patients based on their performance status, disease site, patient preference, and risk/benefit balance.

## 3. Results

A total of 36 (15%) of the 227 female CUP patients were diagnosed with SCCUP (Table 1).

Table 2 shows the characteristics of SCCUP patients, with a median age of 59.9 years (range: 31–90) and a favorable performance status of 0 (61.1%) or 1 (38.9%). The median follow-up was 25.9 months (1–112 months), and the PFS for SCCUP cases was 24.7 months (1–112 months). Of the cases, 22 were single-area SCCUP, and 14 were multi-area. The unfavorable subset, the multi-area SCCUP cases, were treated systemically with anticancer agents. 

Details of the 16 cases in the favorable subset are shown in Table 3. In the favorable subset, surgical excision, local radiotherapy, and a combination of surgery and radiation formed the basis of treatment. 2 cases died within a year of diagnosis: case 1 due to a giant tumor arising in an elderly patient, and case 6 due to laryngeal edema caused by radiation. The other cases had a good prognosis with local treatment.

**Figure 2:**
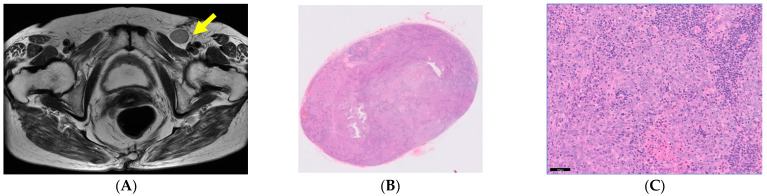
A case of single-area SCCUP classified in the favorable subset. Yellow arrow showed a metastatic tumor in the left inguinal lymph node. (**A**) a metastatic tumor in the left inguinal lymph node. (**B**,**C**) Histological section showing a metastatic tumor in the left inguinal lymph node. (hematoxylin–eosin stain, (**B**): ×4, (**C**): ×20).

Case 2, an 80-year-old woman with a left inguinal mass, was classified as a favorable subset and treated in the gynecology department. Computed tomography showed a metastatic tumor in the left inguinal lymph node (Figure 2A: yellow arrow), and a histopathological examination revealed SCC (Figure 2B,C). No other primary cancer was found on close systemic examination, and only left inguinal lymph node dissection was performed. This case was free of recurrence for 6 years after treatment.

Details of the six cases of single-area SCCUP in the unfavorable subset are shown in Table 4. In the unfavorable subset of six patients of single area not limited to one area, two patients died after systemic chemotherapy, and four patients survived after local treatment only. Multiple distant lung, liver, and lymph node metastases developed, leading to death, in Cases 3 and 5.

Among the unfavorable subset, 14 patients with multiple lesions were all treated with systemic chemotherapy, but 8 patients died.

**Figure 3:**
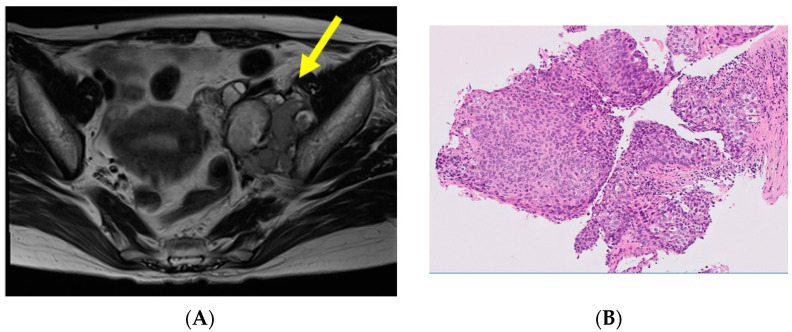
A case of single-area SCCUP classified into the unfavorable subset. Metastatic tumor invasion of the iliopsoas muscle, compression of the left external iliac vein (yellow arrow). (**A**) A CT scan revealed tumor invasion of the iliopsoas muscle, compression of the left external iliac vein. (**B**) Histological section diagnosed SCC. (hematoxylin–eosin stain, ×10).

Case 4, a 48-year-old woman, was classified into an unfavorable subset and treated in the gynecology department; a CT scan revealed tumor invasion of the iliopsoas muscle, compression of the left external iliac vein (Figure 3A), and persistent right leg edema. A CT-guided needle biopsy diagnosed SCC (Figure 3B), and a systemic search including gynecologic cancer screening, gastrointestinal endoscopy, and laryngoscopy failed to detect its primary tumor. The serum SCC antigen level was elevated, at 33.0 ng/mL (reference value: 0–1.5 ng/mL). Concurrent chemoradiotherapy (CCRT) treatment with weekly cisplatin (40 mg/m^2^) was administered in this case, and no recurrence was found at 1 year and 5 months after the primary treatment.

## 4. Discussion

CUP is an extremely rare disease, with around half of patients having disseminated disease and a poor prognosis. Comprehensive reports on the prognosis of SCCUP with local treatment, however, have not been published to date. In the present review, the efficacy of local treatment was found in single-area SCCUP for women.

Previous reports have presented SCCUP in approximately 5% of CUP cases [5,6], while, in this study, SCC accounted for 15% of CUP cases and was much rarer than adenocarcinoma. SCC occurring in the pelvic region includes cervical, vaginal, and vulvar cancers as well as anal and penile cancers as primary sites. Although the primary site of the cancer is unknown, the incidence of SCC is likely higher than previously reported because all the patients in this study were female, and gynecological cancers occur more frequently.

Categorizing SCCUP cases by whether they were single- or multi-area, the localized case was relatively common, with 22 single-area cases and 14 multi-area cases (Table 2). As described above, single-area SCCUP cases can be further divided into two subsets: favorable and unfavorable [10]. Among the favorable subset that is only confined to the neck and inguinal lymph node regions, local treatment is recommended instead of systemic treatment [9]. Hemminki et al. reviewed the prognosis and found that the location of the detected lymph nodes had a significant impact on survival. The lowest hazard ratio (HR) detected by site and histological type was the SCC in the inguinal region [11]. A patient in case 2 (Figure 2) underwent inguinal lymph node resection via the preferred approach, resulting in long-term survival.

Lymph node metastases, except for the inguinal and neck lymph nodes, were not included in the favorable subset. No standard treatment has been established for an unfavorable subset of single-area CUP. Due to the radiosensitivity of SCC, we hypothesized that local treatment such as radiation or surgery might be effective even in the unfavorable subset if SCCUP were confined to a single area. In this study, four patients in the unfavorable subsets survived (Table 4), indicating that local treatment may be effective for single-area SCCUP.

Metastases of SCC are found in the head and neck region, while solitary pelvic metastasis is rare [12,13]. Pelvic retroperitoneal metastases are commonly detected in the uterine cervical malignancy, genitourinary tract malignancy, and immature cystic teratomas of the ovaries [9]. Case 4 (Figure 3) was an unfavorable subset, with SCCUP in the retroperitoneum directly invading the iliopsoas and psoas major muscles. Surgery was considered highly invasive, and CCRT was performed as in gynecologic cancers, since SCC is radiosensitive and shows good response rates to cisplatin-based regimens. The most important prognostic factor for SCC has been reported to be the complete surgical removal of the neoplasm [14,15]. Hemminki et al. reported that pelvic or intra-abdominal lymph node metastases were the most fatal [11], and the long-term prognosis of Case 4 requires further follow-up.

One of the problems in the treatment of CUP is the lack of clinical specialties. In recent years, a department of medical oncology has been established, and the concept of CUP has gradually become more widespread. However, many hospitals do not have oncology departments, and treatment is often provided by various departments, depending on the site where the CUP occurred. In fact, in our study, departments other than Medical Oncology, such as Gynecology, Orthopedic Oncology, and Gastroenterology, also provided treatment (Table 3 and Table 4). Therefore, these departments need to be familiar with the concept of CUP and its treatment strategy in order to provide appropriate treatment. Gynecology departments often treat SCC with pelvic and abdominal lymphadenopathy, such as in the case reported here, with radiotherapy and chemoradiotherapy as effective options. Based on the findings of this study, radiotherapy may provide a better prognosis.

Chemotherapy is currently the most effective treatment for CUP [10], while the ideal chemotherapy regimen remains uncertain. Platinum combination chemotherapy shows a response rate of approximately 25–35%, and CBDCA + PTX combination therapy is currently the most frequently used regimen in clinical practice [16,17,18]. The NCCN Practice Guidelines recommend administering platinum-containing chemotherapy to patients in the unfavorable subset as long as their general condition is maintained [7]. In Case 5, an unfavorable subset, the tumor size was large, and CBDCA + PTX combination therapy was performed before CCRT, resulting in tumor shrinkage. The immune profile of CUP is similar to that of cancer types that respond to immune checkpoint inhibitors (ICIs), and the potential for ICI therapy is expected [19,20]. In a phase II study (NivoCUP study) investigating the efficacy of nivolumab in patients with CUP who received at least one line of systemic chemotherapy, the objective response rate was 22.2% [95% confidence interval (CI), 11.2% to 37.1%] and the median progression-free survival and overall survival were 4.0 months (95% CI, 1.9–5.8 months) and 15.9 months (95% CI, 8.4–21.5 months), respectively [21]. The NivoCUP study included 11 previously untreated patients and showed clinical benefits. This study demonstrated the potential of nivolumab to become a new standard treatment for CUP as a first- or second-line regimen.

There are several limitations to this study. CUP is a rare disease, with SCC accounting for only 15% of these cases. Therefore, it is very difficult to determine the frequency of occurrence, the biological behavior of the tumor, and the current treatments available. In addition, most of the manuscripts published to date have been case reports, and there have been no reports that have examined the treatment outline and prognosis of SCCUP in detail. It is significant that this retrospective study was conducted at the hospital with the largest number of patients treated for SCCUP in Japan, across different departments. Recently, a department of medical oncology has been established to treat rare diseases such as CUP. We hope that these departments will report the accumulated data on the treatment of rare cases in the future, and that they will elucidate the biological behavior of tumors and develop treatments that lead to a better prognosis.

## 5. Conclusions

The present study describes the possibility of regional treatment for SCCUP in women. Single-area SCCUP is expected to be radiosensitive, and complete surgical excision and local radiotherapy are effective options. However, the tumor heterogeneity and the limited number of cases make it difficult to determine the optimal treatment. We presented several treatment options for female SCCUP in this study.

## Figures and Tables

**Table 1:** Pathological diagnosis of CUP in women (n = 227).

Pathological Type	Cases (n)	(%)
Adenocarcinoma	141	62.1
Squamous cell carcinoma	36	15.9
Poorly/undifferentiated carcinoma	19	8.3
Neuroendocrine carcinoma	15	6.6
Sarcoma	7	3.0
Unknown	9	4.0

**Table 2:** Clinical features of SCCUP in women (n = 36).

		Case (n)	(%)
Age	–49	9	25.0
	50–69	17	47.2
	70–	10	27.8
PS	0	22	61.1
	1	14	38.9
Lesion	Single-area	22	61.1
	Multi-area	14	38.9
Category	Favorable subset	16	44.4
	Unfavorable subset	20	55.6

PS: performance status.

**Table 3:** Clinical course of single-area SCCUP classified in the favorable subset.

Case	Year	PS	Lesion	Therapy	Prognosis	Department of Treatment
1	80	1	Neck LN	Palliative RT	DOD 4 M	Radiation Therapy
2	80	0	Inginal LN	Inginal LND	NED	Gynecology
3	79	0	Neck LN	LND	NED	Medical Oncology
4	77	0	Neck LN	LND + RT	NED	Orthopedic Surgery
5	76	0	Neck LN	LND	NED	Orthopedic Surgery
6	73	0	Neck LN	RT	DOD 10 M Laryngeal edema	Orthopedic Surgery
7	72	0	Neck LN	CCRT	NED	Medical Oncology
8	72	0	Neck LN	RT	NED	Medical Oncology
9	68	0	Neck LN	RT	NED	Orthopedic Surgery
10	68	0	Neck LN	NAC + CCRT	NED	Orthopedic Surgery
11	68	0	Neck LN	LND	NED	Orthopedic Surgery
12	68	0	Neck LN	LND + RT	NED	Orthopedic Surgery
13	66	0	Neck LN	LND + RT	NED	Orthopedic Surgery
14	65	0	Neck LN	LND + RT	NED	Orthopedic Surgery
15	54	0	Neck LN	LND + tonsillectomy	NED	Medical Oncology
16	50	0	Neck LN	LND	NED	Orthopedic Surgery

LND: lymph node dissection, RT: radiotherapy, CCRT: concurrent chemoradiotherapy; NAC: neoadjuvant chemotherapy, NED: no evidence of disease, DOD: dead of disease.

**Table 4:** The clinical course of single-area SCCUP classified in the unfavorable subset.

Case	Year	PS	Lesion	Therapy	Prognosis (Year/Months)	Department of Treatment
1	79	0	Iliac muscle~ psoas major muscle	CCRT	Death from gastric cancer	Gastroenterology
2	55	0	Rectum	Ope	NED 2y1m	Gastroenterology
3	53	1	Pelvic bone	Chemo + RT	DOD 3y8m	Orthopedic Oncology
4	48	0	Lt pelvic lymph node~ psoas major muscle	CCRT	NED 1y5m	Gynecology
5	39	0	Rt pelvic lymph node	Chemo + CCRT	DOD 2y11m	Medical Oncology
6	31	0	Lt pelvic lymph node	CCRT	NED 6y6m	Gynecology

PS: performance status, CCRT: concurrent chemoradiotherapy, Ope: operation, Chemo: chemotherapy, RT: radiotherapy, NED: no evidence of disease, DOD: dead of disease.

## Data Availability

The data presented in this study are available on request from the corresponding author.
